# Lint percentage and boll weight QTLs in three excellent upland cotton (*Gossypium hirsutum*): ZR014121, CCRI60, and EZ60

**DOI:** 10.1186/s12870-023-04147-5

**Published:** 2023-04-05

**Authors:** Hao Niu, Meng Kuang, Longyu Huang, Haihong Shang, Youlu Yuan, Qun Ge

**Affiliations:** 1 State Key Laboratory of Cotton Biology, Key Laboratory of Biological and Genetic Breeding of Cotton, Institute of Cotton Research, The Ministry of Agriculture, Chinese Academy of Agricultural Sciences, Anyang, 455000 Henan China; 2 Zhengzhou Research Base, State Key Laboratory of Cotton Biology, Zhengzhou University, Zhengzhou, 450001 Henan China

**Keywords:** Upland cotton (*Gossypium hirsutum* L.), Lint percentage, Boll weight, Quantitative trait locus (QTL), Candidate gene

## Abstract

**Background:**

Upland cotton (*Gossypium hirsutum* L.) is the most economically important species in the cotton genus (*Gossypium* spp.). Enhancing the cotton yield is a major goal in cotton breeding programs. Lint percentage (LP) and boll weight (BW) are the two most important components of cotton lint yield. The identification of stable and effective quantitative trait loci (QTLs) will aid the molecular breeding of cotton cultivars with high yield.

**Results:**

Genotyping by target sequencing (GBTS) and genome-wide association study (GWAS) with 3VmrMLM were used to identify LP and BW related QTLs from two recombinant inbred line (RIL) populations derived from high lint yield and fiber quality lines (ZR014121, CCRI60 and EZ60). The average call rate of a single locus was 94.35%, and the average call rate of an individual was 92.10% in GBTS. A total of 100 QTLs were identified; 22 of them were overlapping with the reported QTLs, and 78 were novel QTLs. Of the 100 QTLs, 51 QTLs were for LP, and they explained 0.29–9.96% of the phenotypic variation; 49 QTLs were for BW, and they explained 0.41–6.31% of the phenotypic variation. One QTL (*qBW-E-A10-1*, *qBW-C-A10-1*) was identified in both populations. Six key QTLs were identified in multiple-environments; three were for LP, and three were for BW. A total of 108 candidate genes were identified in the regions of the six key QTLs. Several candidate genes were positively related to the developments of LP and BW, such as genes involved in gene transcription, protein synthesis, calcium signaling, carbon metabolism, and biosynthesis of secondary metabolites. Seven major candidate genes were predicted to form a co-expression network. Six significantly highly expressed candidate genes of the six QTLs after anthesis were the key genes regulating LP and BW and affecting cotton yield formation.

**Conclusions:**

A total of 100 stable QTLs for LP and BW in upland cotton were identified in this study; these QTLs could be used in cotton molecular breeding programs. Putative candidate genes of the six key QTLs were identified; this result provided clues for future studies on the mechanisms of LP and BW developments.

**Supplementary Information:**

The online version contains supplementary material available at 10.1186/s12870-023-04147-5.

## Background

Cotton (*Gossypium*) is an economically important natural fiber plant. Upland cotton (*Gossypium hirsutum*) is the most widely cultivated cotton variety, accounting for approximately 95% of global cotton production [1, 2]. Increasing the yield of upland cotton remains the main objective of this important cash crop worldwide. Cotton yield is typically affected by several complex quantitative traits, including the boll number (BN), lint percentage (LP), boll weight (BW), seed index (SI) and lint index (LI) [3]. These yield component traits are controlled by genetic factors and are affected by environmental factors; they are also genetically related to each other [3–5]. LP is an economically important index for cotton cultivars with the highest heritability [6]. Because LP is a key contributor to lint yield and is easy to measure, selection for increasing LP has become an important approach for enhancing lint yield [7, 8]. Numerous studies have shown that cotton yield mainly depends on LP, BW, and BN, and these traits have been positively selected in cultivated cotton throughout the domestication process [9–15].

Because cotton breeding requires excellent germplasm, a large amount of germplasm resources have been preserved and improved in China, such as many high LP cultivars/lines [16–18]. Many interspecific introgressive lines (ILs) or chromosome segment introgression lines (CSILs) have been obtained by crosses between *G. hirsutum* and *Gossypium barbadense* [19, 20]; some of these lines have high LP and BW [21]. Many new germplasm resources and cultivars have been successfully bred [22–26]. Our lab has also bred a set of advanced cotton lines/cultivars, such as the parents used in this study.

The identification of stable and effective quantitative trait loci (QTLs) is prerequisites for cotton molecular breeding. From 1998 to 2015, a total of 327 QTLs for LP and 170 QTLs for BW were identified on different chromosomes through meta-QTL analysis [27]. Following the release of the cotton genome sequence, the number of discovered QTLs is rapidly increasing via genome-wide association study (GWAS) or linkage mapping [28–30]. For example, structural variations have been explored by resequencing 1,081 *G. hirsutum* accessions, and 446 structural variations are significantly associated with seven traits, including 21 with LP and 17 with BW [31]. Genetic linkage analysis and association analysis (AS, or GWAS) are the two major approaches for identifying QTLs in crops. Many high-density genetic linkage maps and association maps for cotton have been published. For example, more than 17 crosses or populations of upland cotton have been used to construct genetic maps, including crosses of Yumian1 × T586 [4, 32, 33], Yumian1 × Zhongmiansuo35 [1], NC05AZ06 × NC11-2091 [34], DH962 × Jimian5 [35–37], Zhongmiansuo12 (ZMS12) × 8891 [4], (Simian3 × Sumian12) × (Zhong4133 × 8891) [3], Baimian1 × TM-1 [38, 39], Xiangzamian2 [40, 41], HS46 × MARCABUCAG8US-1–88 [42, 43], and CCRI35 × Nan Dan Ba Di Da Hua (NH) [44]. One high-density bin linkage map contains 6,187 bin markers spanning 4,478.98 cM with an average distance of 0.72 cM [18]. Different types of GWAS, including single-locus-GWAS (SL-GWAS), multi-locus GWAS (ML-GWAS), and restricted two-stage, multi-locus, and multi-allele GWAS (RTM-GWAS) approaches, have been used to identify quantitative trait nucleotides (QTNs) for LP and BW in several cotton accessions. More than 16 association maps and many candidate genes for agronomic traits have been reported [5, 8, 10, 12, 45–48]. For example, 86 single-nucleotide polymorphism linkage disequilibrium block (SNPLDB) loci for LP and 70 SNPLDB loci for BW have been identified from 315 cotton accessions using RTM-GWAS [12]. A total of 719 upland cotton accessions have been screened by GWAS using the cottonSNP63K array, and 62 identified single nucleotide polymorphism (SNP) loci were significantly associated with different traits; a total of 689 candidate genes were screened, and 27 of them contain at least one significant SNP, including three for LP and six for BW [5].

Although the inheritance, QTLs and candidate genes of LP and BW in upland cotton have been widely studied, only a few of the studied QTLs have been used in the molecular breeding of cotton via marker-assisted selection (MAS) [49, 50]. One of the reasons is that the identified QTLs are unstable in multiple-environments and only explain little phenotypic variance. Consequently, mining stable, effective LP and BW-related QTLs or QTNs would greatly aid cotton molecular breeding. We have previously bred the excellent cotton lines ZR014121 and EZ60 and the cultivar CCRI60. Here, we identified stable, effective LP and BW-related QTLs to aid the utilization of the germplasm resources in cotton breeding.

## Results

### Phenotypic variation in LP and BW

**Fig. 1:**
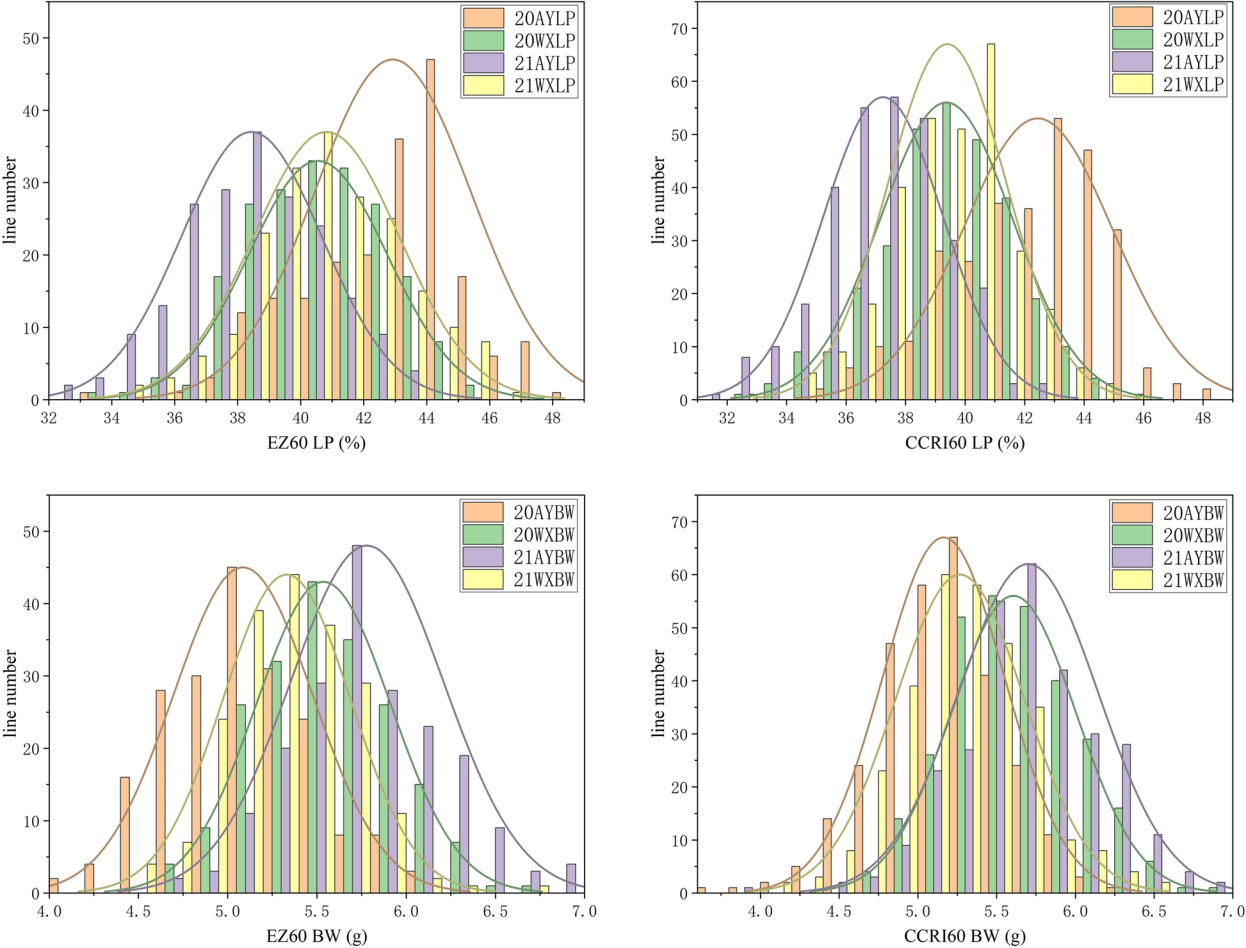
The histograms of the LP and BW in P-EZ60 (EZ60) and P-CCRI60 (CCRI60) in Anyang and Weixian in 2020 and 2021

We evaluated two yield-related traits LP and BW, in the two recombinant inbred line (RIL) populations under four environments in 2020 and 2021. The LP and BW ranged from 32.56% to 48.26% and from 4.09 to 6.93 g in P-EZ60, respectively (Table 1); LP and BW ranged from 31.57% to 48.02% and from 3.68 to 6.83 g in P-CCRI60, respectively (Table 2). All of the absolute skewness values of LP and BW were less than 1.0. The distributions of the LP and BW in the four experimental environments were normal. This suggests that LP and BW are polygenic traits, and the data could be used to map QTLs (Fig. 1). LP and BW exhibited high degrees of phenotypic variation. The coefficient of variation for each trait was relatively consistent among the different environments, suggesting that LP and BW were significantly affected by the environment, and the effect on BW (average 7.16 in P-EZ60; 7.55 in P-CCRI60) was greater than that on LP (average 5.69 in P-EZ60; 5.51 in P-CCRI60) (Tables 1, and 2).

**Table 1:** Statistical analysis of the BW and LP in P-EZ60

environment	trait	parents		population									
		ZR014121	EZ60	RILs	range	Minimum value	Maximum value	mean value	standard deviation	variance	skewness	kurtosis	coefficient of variation
20AY	LP	39.47	41.87	199	14.42	33.83	48.26	42.92	2.5	6.24	-0.547	0.161	5.82
	BW	4.43	5.17	199	2.02	4.09	6.11	5.09	0.39	0.153	0.152	-0.196	7.69
20WX	LP	38.91	40.29	199	12.09	33.57	45.67	40.55	2.2	4.853	-0.198	-0.145	5.43
	BW	5.1	5.35	199	2.04	4.6	6.64	5.54	0.38	0.142	0.145	-0.216	6.8
21AY	LP	36.35	39.69	199	10.93	32.56	43.49	38.44	2.24	5.013	-0.105	-0.29	5.83
	BW	5.21	6.13	199	2.28	4.65	6.93	5.78	0.43	0.185	0.207	0.123	7.43
21WX	LP	38.54	40.83	199	11.96	34.33	46.29	40.81	2.32	5.399	-0.014	-0.084	5.69
	BW	4.96	5.73	199	2.23	4.41	6.63	5.33	0.36	0.129	0.147	0.415	6.73

**Table 2:** Statistical analysis of the BW and LP in P-CCRI60

environment	trait	parents		population									
		ZR014121	CCRI60	RILs	range	Minimum value	Maximum value	mean value	standard deviation	variance	skewness	kurtosis	coefficient of variation
20AY	LP	39.47	41.48	299	13.02	35	48.02	42.44	2.51	6.318	-0.468	-0.264	5.92
	BW	4.43	5.15	299	2.54	3.68	6.23	5.16	0.39	0.15	-0.37	0.732	7.5
20WX	LP	38.91	39.33	299	12.18	32.66	44.84	39.37	2.23	4.972	-0.305	0.095	5.66
	BW	5.1	5.65	299	2.12	4.71	6.83	5.61	0.4	0.158	0.22	-0.325	7.09
21AY	LP	36.35	37.02	299	11	31.57	42.57	37.24	2.01	4.037	-0.11	-0.067	5.39
	BW	5.21	5.62	299	2.91	3.92	6.83	5.7	0.44	0.198	-0.172	0.478	7.8
21WX	LP	38.54	39.24	299	13.38	32.15	45.52	39.4	2	3.992	-0.135	0.391	5.07
	BW	4.96	5.49	299	2.55	4	6.55	5.27	0.41	0.168	0.093	0.325	7.79

The correlations between LP and BW of all the RILs in the four environments were analyzed separately. Generally, LP and BW were significantly negatively correlated in P-EZ60 and P-CCRI60, and the coefficients ranged from -0.098 to -0.340, which suggested that it was difficult to improve LP and BW synchronously (Tables 3, and 4). Because the cotton field was waterlogged in Anyang in 2021, the LP and BW were affected to some extent, but the phenotypic data met the requirements for GWAS (Fig. 1). Analysis of variance (ANOVA) showed that there were highly significant differences among the accessions and environments for the two traits of two populations (Table 5). It indicated that LP and BW were significantly influenced by the accessions and planting environments.

**Table 3:** Correlation analysis between BW and LP in P-EZ60 in Anyang in 2020 and 2021

	20AYLP	20WXLP	21AYLP	21WXLP
20AYBW	-0.107^*^			
20WXBW		-0.247^**^		
21AYBW			-0.191^**^	
21WXBW				-0.340^**^

^**^ Represents significance at the *P* < 0.01 level (two-tailed)

^*^ Represents significance at the *P* < 0.05 level (two-tailed)

**Table 4:** Correlation analysis between BW and LP in P-CCRI60 in Anyang in 2020 and 2021

	20AYLP	20WXLP	21AYLP	21WXLP
20AYBW	-0.098^*^			
20WXBW		-0.247^**^		
21AYBW			-0.185^**^	
21WXBW				-0.234^**^

^**^ Represents significance at the *P* < 0.01 level (two-tailed)

^*^ Represents significance at the *P* < 0.05 level (two-tailed)

**Table 5:** Analysis of variance for the two traits of two populations

population	trait	Source	SS	df	MS	F	*P*-value
P-EZ60	LP	Accessions	6714.21	190	35.34	5.58	0.000
		Environments	3856.09	3	1285.36	173.44	0.000
	BW	Accessions	151.92	190	0.80	3.24	0.000
		Environments	110.12	3	36.71	151.13	0.000
P-CCRI60	LP	Accessions	7788.48	290	26.86	3.32	0.000
		Environments	8105.79	3	2701.93	388.89	0.000
	BW	Accessions	216.49	290	0.75	2.85	0.000
		Environments	124.62	3	41.54	154.30	0.000

### *SNP quality control and *in silico* mapping*

According to the high-throughput whole-genome sequencing data of upland cotton (Nanjing Agricultural University), a liquid SNP array with 10 K SNPs was developed. The two RIL populations of P-CCRI60 and P-EZ60, including their parents, were genotyped by genotyping by target sequencing (GBTS) (Table S1). The total number of samples was 500. The average call rate of a single locus was 94.35%, and the average call rate of an individual was 92.10%. The results of the genotype control are shown in supplementary table 2 (Table S2). The BLAST alignment tool was used to analyze the probe sequences of SNPs against the *G. hirsutum* TM-1 genome sequence [28, 51], and a total of 8,348 genotyped high-quality SNPs across the 500 samples were used in association mapping.

### Genome-wide association studies

**Fig. 2:**
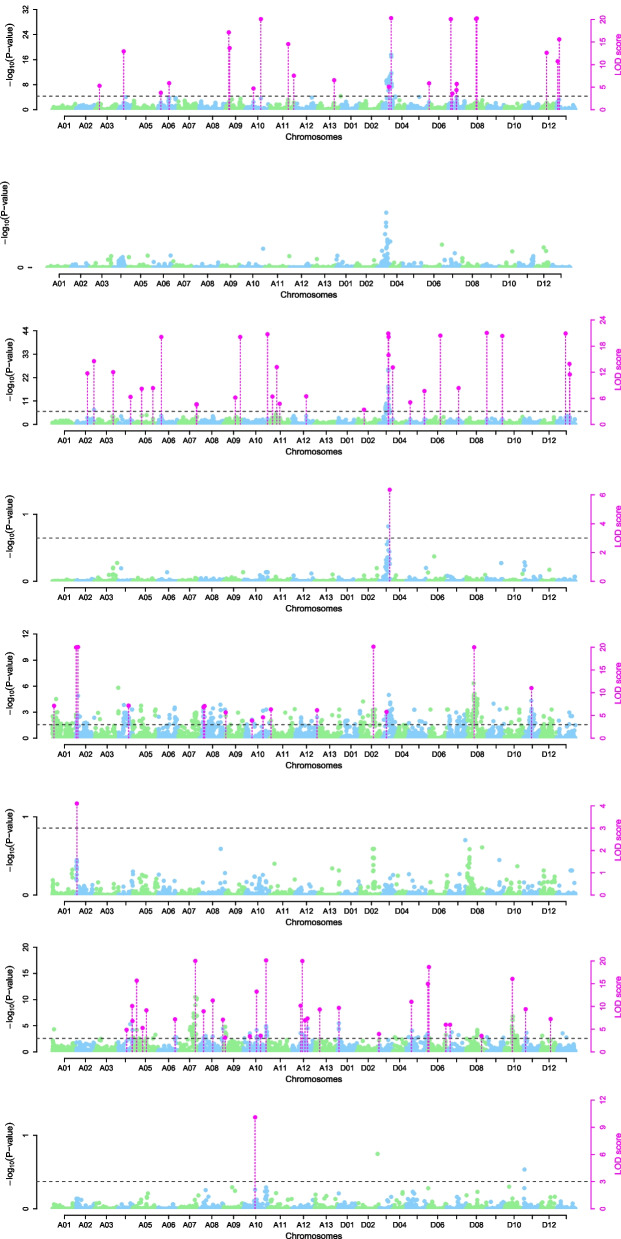
Manhattan-plots of LP and BW using the genetic model 3VmrMLM. X-axes are cotton chromosomes. Y-axes on the left side report -log10 P-values of the main-effect QTNs, which were obtained from single-marker genome-wide scans for all the markers in the first step of 3VmrMLM; Y-axes on the right side report LOD scores, which were obtained from likelihood ratio tests for significant and suggested QTNs, with a threshold of LOD = 3.0 (dashed line) in the second step of 3VmrMLM. These LOD scores are indicated by points with straight lines

We used the genetic model of 3VmrMLM to detect QTNs for LP and BW × environment interactions (Fig. 2). A total of 104 stable quantitative trait nucleotides (QTNs) on 26 chromosomes were identified as significantly associated with LP and BW (Table S3). Following other similar studies [47], we defined the flanking 200-Kb regions of QTNs as an initial QTL and merged the overlapping QTLs to obtain the final QTLs. In the end, 100 stable QTLs were detected; 51 of them were for LP and 49 were for BW, including three QEIs, one for LP and two for BW, which could be identified in the four environments (Table S4). A total of 20 stable QTLs, 14 for LP and 6 for BW, were identified in EZ60, including one QEI for BW that could be identified in the four environments; 33 stable QTLs, 18 for LP and 15 for BW, were identified in CCRI60, including one QEI for LP that could be identified in the four environments; and 47 stable QTLs were identified in ZR014121, 19 for LP and 28 for BW, including one QEI for BW that could be identified in the four environments (Table S4). One QTL in chromosome A10, *qBW-E-A10-1*, was identified in both populations. Among the 100 QTLs, 22 QTLs, 9 for LP and 13 for BW, were overlapping with the reported QTLs (Table S5); 78 QTLs, 42 for LP and 36 for BW, were novel (Table S6).

The QTLs explained 0.29–9.96% of the phenotypic variations in LP or BW. In P-EZ60, the novel QTLs associated with LP explained 0.47–8.67% of the phenotypic variation, and the novel QTLs associated with BW explained 0.91–6.31% of the phenotypic variation. In P-CCRI60, the novel QTLs associated with LP explained 0.29 –9.96% of the phenotypic variation, and the novel QTLs associated with BW explained 0.36–3.02% of the phenotypic variation.

**Fig. 3:**
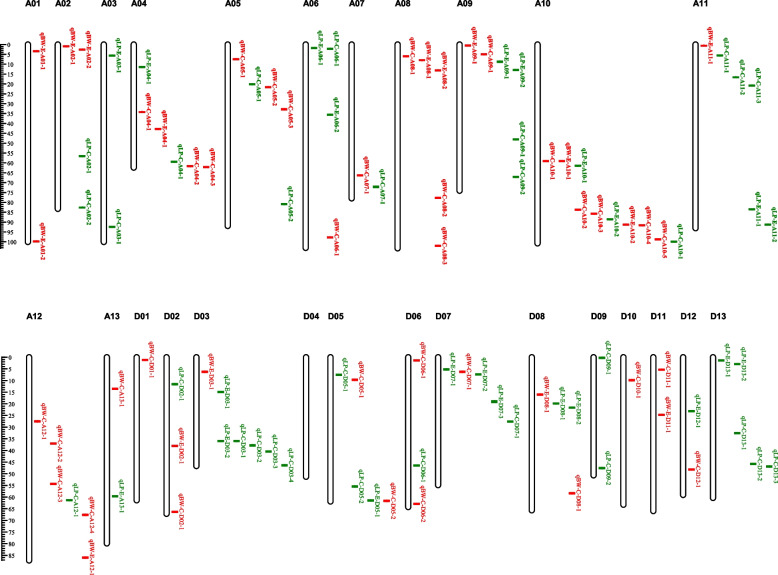
A physical map of QTLs for LP and BW from the two RIL populations. The green letters are QTLs for LP, and the red letters are QTLs for BW. The scale on the left is in Mb

In sum, a total of 51 QTLs related to LP were detected in this study, including 14 in EZ60, 18 in CCRI60, and 19 in ZR014121; 28 QTLs were in the At subgenome, and 27 QTLs were in the Dt subgenome, indicating that LP-related QTLs were evenly distributed in the At and Dt subgenomes. A total of 49 QTLs related to BW were detected, including 6 in EZ60, 15 in CCRI60, and 28 in ZR014121; 34 QTLs were in the At subgenome, and 15 QTLs were in the Dt subgenome, indicating that the QTLs related to BW were mainly distributed in the At subgenome. There were two QEIs, which were located on chromosomes A02 and A10 (Fig. 3).

### Candidate genes in the regions of the six key QTLs

To identify candidate genes of key QTLs, six QTLs were selected, including three QEIs, the common QTL *qBW-E-A10-1* that was mapped in both populations and two important QTLs (*qLP-E-D03-2* and *qLP-C-D03-2*). The three QEIs were QTLs that were stable in the four environments (Table S7). A total of 108 putative candidate genes in the regions of the six key QTLs in multiple environments were identified, including genes that were positively related to LP and BW, such as the genes involved in gene transcription, protein synthesis, calcium signaling, phytohormone synthesis and signaling, and fiber synthesis-related polysaccharide metabolism (Table S6).

**Fig. 4:**
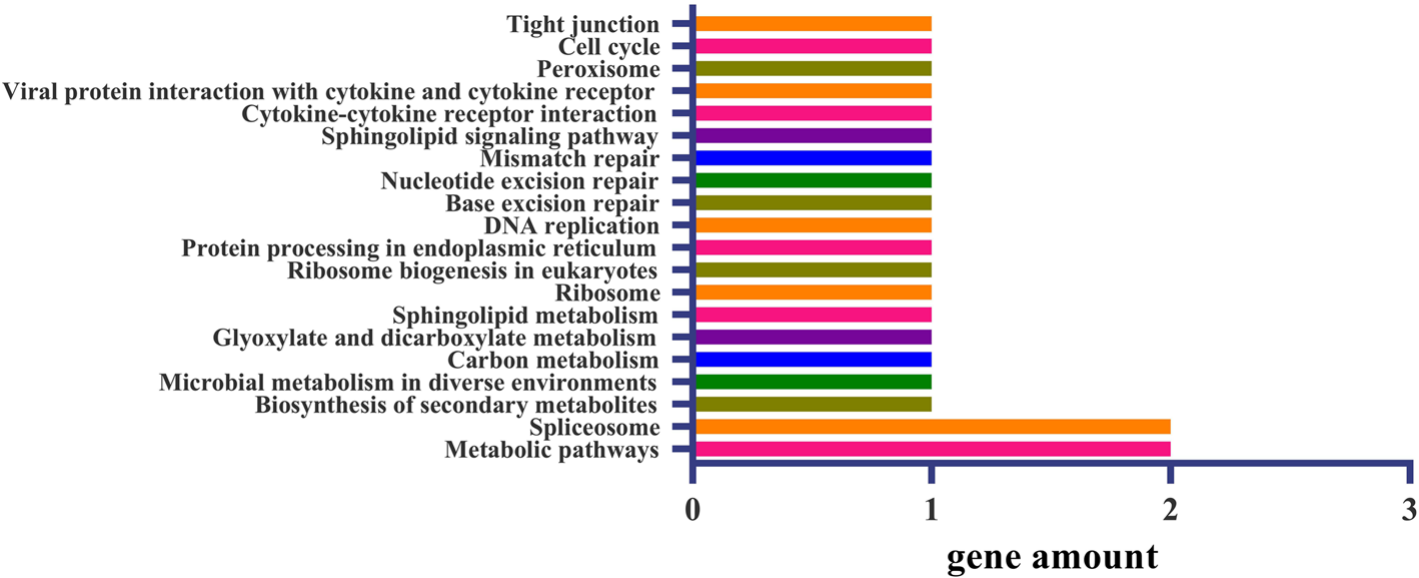
A histogram of candidate genes enriched in different KEGG pathways for LP. The x-axis indicates the number of candidate genes. The y-axis represents biological processes. The details are listed in Table S9

**Fig. 5:**
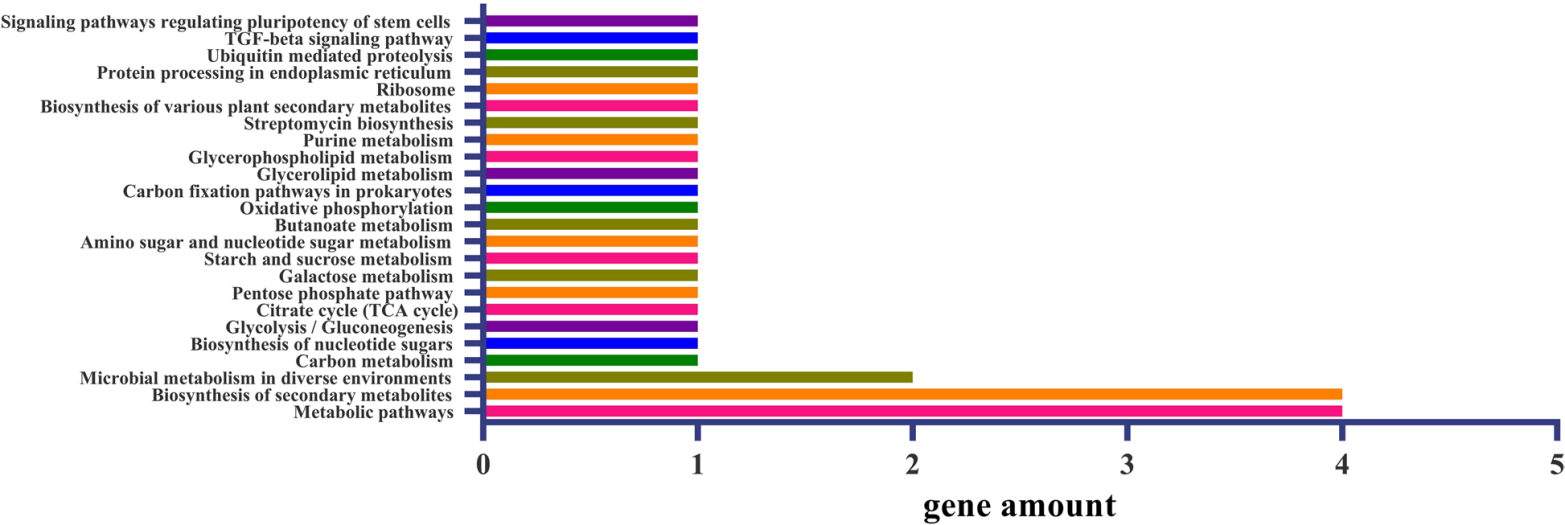
A histogram of candidate genes enriched in different KEGG pathways for BW. The x-axis indicates the number of candidate genes. The y-axis represents biological processes. The details are listed in Table S10

KEGG analysis showed that the 48 genes related to LP were mainly involved in “metabolic pathways” and “spliceosome” (Fig. 4). Eighteen metabolic pathways such as “biosynthesis of secondary metabolites”, “microbial metabolism in diverse environments” and “DNA replication” were also detected. KEGG analysis showed that the 60 genes related to BW were mainly involved in “metabolic pathways” and “biosynthesis of secondary metabolites” (Fig. 5). “Microbial metabolism in diverse environments”, “carbon metabolism,” “glycolysis/gluconeogenesis,” and 19 other metabolic pathways were detected.

### Expression profiles of candidate genes during fiber development

**Fig. 6:**
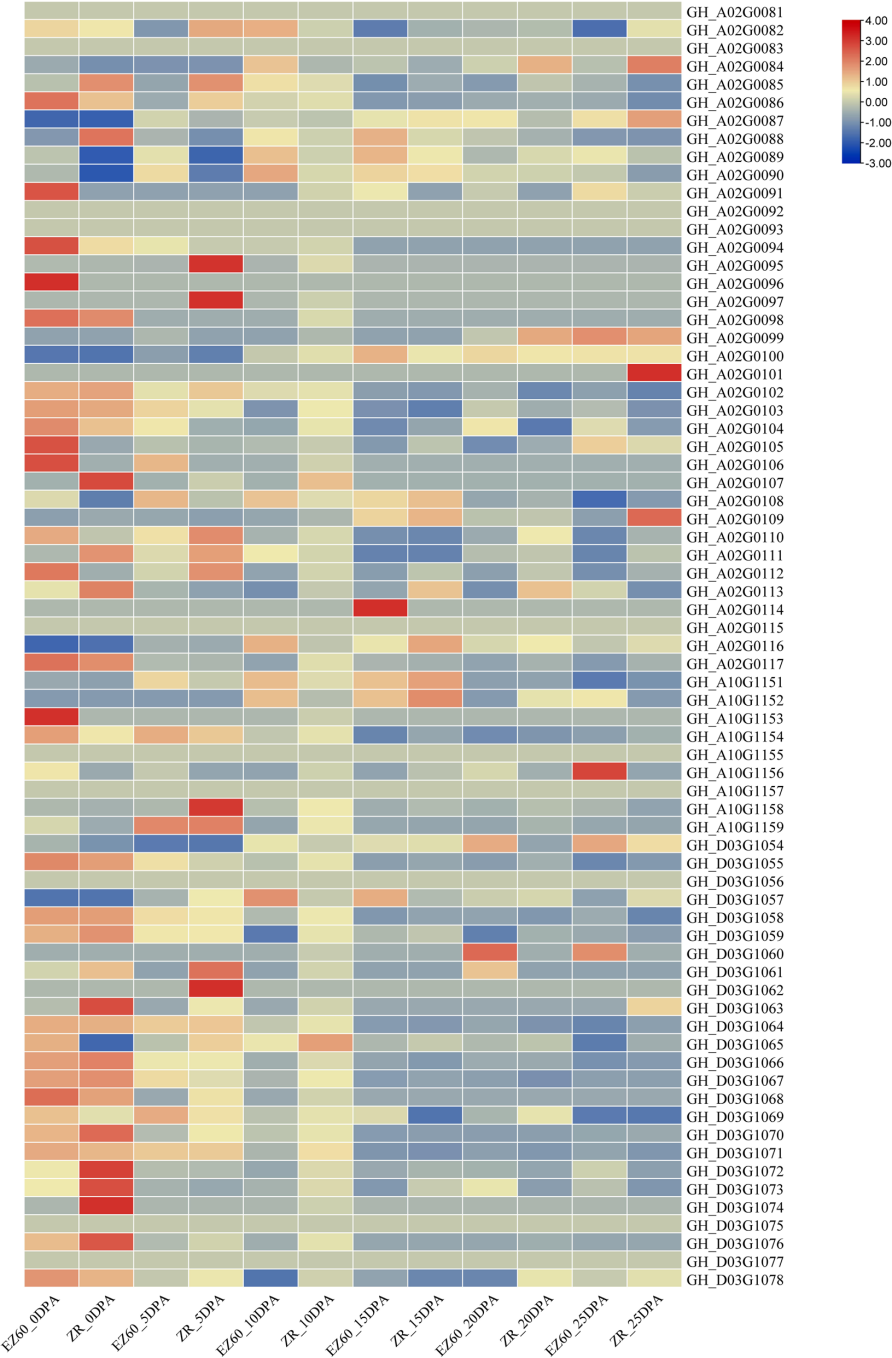
Gene expression profiles of the candidate genes of LP and BW QTLs during fiber development in EZ60 and ZR014121. Each column represents one sample, and rows represent candidate genes. The expression levels of the candidate genes (FPKM) were log2-normalized (i.e., log2(FPKM + 0.01)) and presented in different colours on the scale bar. ZR indicates cotton line ZR014121; DPA indicates days post-anthesis. 0 DPA represents the ovule development stage. 5, 10, 15, 20, and 25 DPA represent the fiber development stages. Detailed information on gene expression is shown in Table S11

Most of the candidate genes associated with LP and BW were differentially expressed in cotton fiber at different developmental stages, and there were differences at expression levels between the high-LP parent EZ60 and the low-LP parent ZR014121 at the same stage (Fig. 6). Among the major candidate genes, *Gh_A02G0096* was only expressed in the ovule developmental stage of EZ60. *Gh_A02G0111* was mainly expressed in both EZ60 and ZR014121 at 0, 5, 10, and 20 days post-anthesis (DPA). Its expression levels were higher in ZR014121 than in EZ60 at 0, 5, and 25 DPA; its expression levels were higher in EZ60 than in ZR014121 at 10 DPA. *Gh_D03G1064* was highly expressed in both EZ60 and ZR014121 at all stages. It was mainly expressed at 0, 5, and 10 DPA, and its expression level in ZR014121 was higher than that in EZ60 at 10 DPA. *Gh_D03G1069* was expressed in both EZ60 and ZR014121 at all stages. Its expression levels were higher in ZR014121 than in EZ60 at 10 and 20 DPA; its expression levels were higher in EZ60 than in ZR014121 at 0, 5, 15, and 25 DPA. *Gh_A02G0106* was significantly highly expressed during the ovule development stage in EZ60, highly expressed at 5 DPA, and weakly expressed at 10 DPA in ZR014121.

### Co-expression of candidate genes

**Fig. 7:**
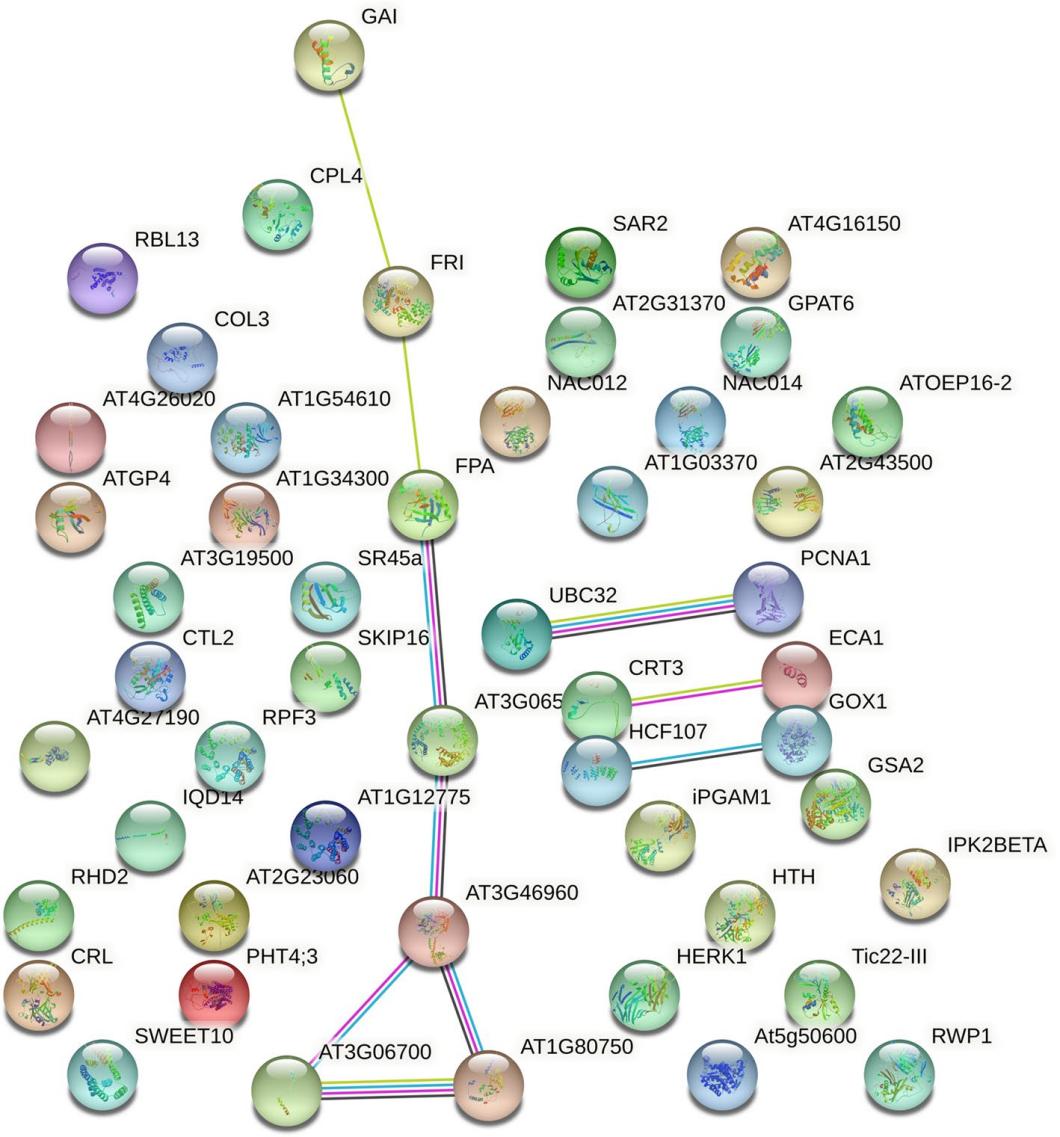
Protein–protein interaction of the candidate genes of the QTLs for LP and BW. Network nodes represent proteins with splice isoforms or post-translational modifications are collapsed, i.e. each node represents all the proteins produced by a single, protein-coding gene locus. Colored nodes: query proteins and first shell of interactors; white nodes: second shell of interactors; Empty nodes: unknown proteins. 3D structure filled nodes: some 3D structures are known or predicted. Edges represent protein–protein associations. Associations are meant to be specific and meaningful (i.e., proteins jointly contribute to a shared function); this does not necessarily mean that they physically bind to each other. Known Interactions, blue: from curated databases; purple: experimentally determined. Predicted Interactions, green: gene neighborhood, red: gene fusions; indigo: gene co-occurrence; Others, yellow: textmining, black: co-expression, light purple: protein homology

**Fig. 8:**
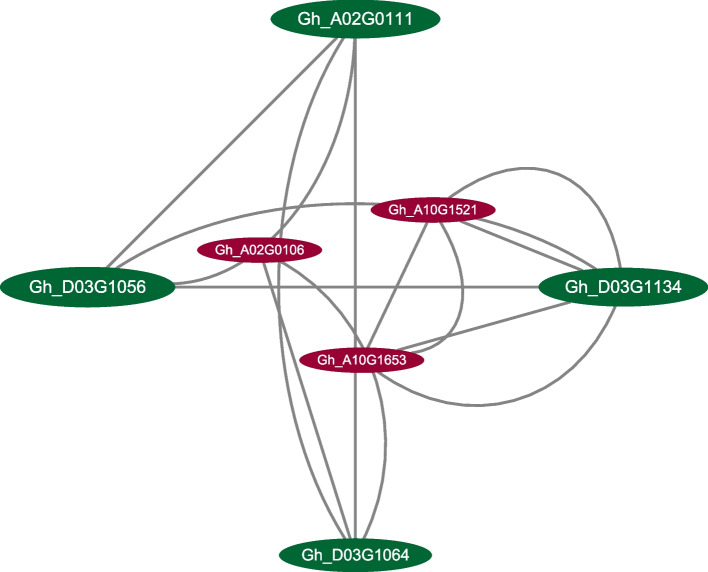
Major gene coexpression network of the candidate genes of the QTLs for LP and BW. Lines indicate co-expression of two linked genes. Network nodes represent genes. The size of the circle shows the betweenness centrality points of the gene. The size of the circle indicates that the gene plays an important role in co-expression. In this graph, genes with higher betweenness centrality points are marked in green and placed in the outer circle, and genes with smaller BC values are marked in red and placed in the inner circle. The three genes in the outer ring, Gh_D03G1056, Gh_D03G1064, and Gh_D03G1134 were candidate genes for LP, and Gh_A02G0111 was a candidate gene for BW

The interaction network of candidate genes associated with LP and BW was investigated by constructing the protein–protein interaction (PPI) network using the STRING database [52] (Fig. 7). Correlations were observed in the expression of the following proteins that appear to comprise a co-expression network: Gh_A02G0111, Gh_D03G1056, Gh_D03G1134, Gh_D03G1064, Gh_A02G0106, Gh_A10G1521, and Gh_A10G1653. Network analysis of the major proteins was carried out using Cytoscape 3.7.2 (Fig. 8). Gh_D03G1056, Gh_D03G1064, Gh_D03G1134, and Gh_A02G0111 played important roles in the network.

PPI analysis indicated that GAI interacted with six other proteins. GAI interacted with FRI; FRI interacted with FPA; FOA interacted with AT1G12775; AT1G12775 interacted with AT3G46960; and AT3G46960, AT3G06700, and AT1G80750 interacted with each other (Fig. 7). There were three groups of co-expressed genes, UBC32 and PCNA1; and CRT3 and ECA1; HCF107 and GOX1. Co-expression analysis of the 108 candidate genes of the six QTLs using Cytoscape 3.7.2 indicated that the seven genes (the same as the result of PPI) were co-expressed, including *Gh_A02G0106* (*GAI*) (Fig. 8).

## Discussion

### A set of new major QTLs for LP and BW that could be used for MAS was obtained

LP and BW are the most important traits in cotton breeding, and they have been widely studied. More than 417 unique QTLs for LP have been identified on 26 chromosomes, including 243 QTLs identified with LOD > 3. More than 60 were stable, major effective QTLs that could be used for MAS [50]. According to the CottonGen Database [53, 54], a total of 1,387 yield QTLs and four yield component trait QTLs have been identified. The numbers of these QTLs are increasing continually. Recently, 34 SNPs corresponding to 22 QTLs for LP, including 13 novel QTLs, were detected from 254 upland cotton accessions via GWAS [55]. Two stable LP QTLs and three BW QTLs were identified in the RIL mapping population derived from the inter-specific cross between *G. hirsutum* cv DS-28 and *G. barbadense* cv SBYF-425 [56]. We also identified one QTL for LP, and nine QTLs for BW from a BC_5_F_3:5_ chromosome segment substitution line population derived from *G. hirsutum* CCRI36 and *G. barbadense* Hai1 [57]. Three QTLs for LP and one QTL for BW were identified from an F_2_ population derived from the *G. hirsutum* × *G. barbadense* cross [58].

In this study, a total of 51 stable QTLs for LP and 49 stable QTLs for BW were identified from three upland cotton lines ZR014121, CCRI60, and EZ60; these QTLs could explain 0.29–9.96% of the phenotypic variation in LP and 0.41–6.31% of the phenotypic variation in BW. A total of 78 of these QTLs were novel. These findings enhance QTL resources that could be used to enhance the yield of cotton; this QTL information will also aid the molecular breeding of cotton cultivars with high yield.

Many studies have shown that the heritability of LP is the highest among all yield component traits in cotton, and the heritability of BW was the lowest among all cotton yield components. Because the heritability of BW is low, environmental factors can have significant effects on BW [6, 59–61]. The results of this study also demonstrate that environmental factors have stronger effects on BW than on LP (Tables 1, and 2). Thus, selection for LP can achieve desired outcomes more efficiently than selection for BW in cotton breeding. Correlations and path analysis among agronomic and technological traits of 16 upland cotton lines indicated that LP was negatively correlated with BW (-0.2668) [62]. Generally, LP and BW are negatively related [50]. In our study, the correlation coefficients between LP and BW ranged from -0.098 to -0.340 (Tables 3, and 4). This indicates that increases in one of these traits limit increases in the other. LP may be the target of direct selection on cotton genotypes with high cotton fiber yield.

Most QTLs for LP and BW explain less than 10% of the phenotypic variation. For example, one study indicates that nine QTLs for LP explain 1.84–13.50% of the observed phenotypic variation; two QTLs for BW explain 6.02–9.50% of the observed phenotypic variation [63]. The QTLs *qLP-C13-1* and *qLP-C25-1* for LP explain 5.77% and 8.87% of the phenotypic variation, respectively [64]. A GWAS of a set of 289 *Gossypium arboreum* chromosome segment ILs in *G. hirsutum* indicates that co-QTLs for LP explain 1.21–10.79% of the phenotypic variation, and co-QTLs for BW explain 1.17–11.56% of the phenotypic variation [65]. Some QTLs for LP identified in this study explained nearly 10% of the phenotypic variation, and all QTLs for BW explained less than 10% of the phenotypic variation (Table S4). These QTLs, especially the major effective QTLs, can be used to breed cotton plants with high yield via MAS.

### Several putative candidates of the six QTLs for LP and BW were identified

Understanding the molecular mechanisms of LP and BW developments is essential for the molecular breeding of cotton plants with high yield, especially via genetic engineering. Many candidate genes of the QTLs for LP and BW have been studied [48–50, 55]. The TIP41-like family protein (*TIP41L*) gene (*GH_A12G0194*) is thought to be the candidate gene of a stable major QTL (*q*(*BW* + *SI*)*-A12-1*) for BW [49]. One gene orthologous to the *Arabidopsis* receptor-like protein kinase gene *HERK1* (*GB_A07G1034*) was predicted to be the candidate gene for LP in *G. barbadense* [48]. Two candidate genes (*Gh_D01G0162* and *Gh_D07G0463*) of QTLs for LP were identified. *Gh_D01G0162* is a homolog of the auxin-responsive GH3 family protein gene, and *Gh_D07G0463* is a homolog of the NADPH/respiratory burst oxidase protein D gene (*RBOHD*) in Arabidopsis [55]. A molecular regulatory network for LP has been proposed based on the functions of the candidate genes of QTLs for LP [50].

In this study, the candidate genes of the six important QTLs for LP and BW were investigated. The QTLs for both traits have candidate genes involved in gene transcription, protein syntheses, signaling, calcium signaling, carbon metabolism, metabolic pathways, and biosynthesis of secondary metabolites, which demonstrates that there are several candidate genes of the QTLs for LP and BW (Figs. 4, and 5; Tables S8, S9, S10). This result is consistent with the findings of previous studies [48, 50, 55, 66, 67]. The difference is that a greater number of candidate genes in QTLs for LP were involved in gene expression processes, and a greater number of candidate genes in QTLs for BW were involved in metabolic pathways. Interaction network analysis of the candidate genes associated with LP and BW indicated that seven candidate genes could form a co-expression network. The candidate gene *Gh_A02G0096* of *qBW-E-A02-1* encodes a homolog of eukaryotic translation initiation factor 2A, and the candidate gene *Gh_D03G1069* of *qLP-E-D03-2* likely encodes a serine/threonine-protein kinase. Their interaction suggests that LP and BW are closely related during development (Figs. 7, and 8). Additional studies are needed to clarify why LP and BW are negatively related.

### Many candidates of the six QTLs are involved in fiber development

The MYB-bHLH-WD40 (including MYB-DEL-TTG and CPC-MYC-TTG) [33, 68] and TCP-HOX-HD [66, 69] regulatory complexes play key roles in cotton fiber development. Phytohormone balance, Ca^2+^ signaling, and ROS also play key roles regulating fiber development [50, 70, 71].

Many candidate genes of the QTLs for LP and BW are involved in various signaling pathways and metabolic processes in this study, such as the transcription factor *bHLH113* gene (*Gh_A02G0095*); Ca^2+^ signaling genes (*Gh_A10G1519*, *Gh_D03G1058*, and *Gh_D03G1266*); protein kinase genes (*Gh_D03G1144*, *Gh_D03G1264*, and *Gh_D03G1069*); GA signaling genes (*Gh_A02G0104* and *Gh_A02G0106*); and ROS metabolism-related genes (*Gh_D03G1138*, *Gh_D03G1063*, and *Gh_D03G1062*) [55] (Table S7). *Gh_D03G1264* encodes a HERK1-like protein [48]. *Gh_A02G0106* is a homolog of *AT1G14920*, that encodes a gibberellin insensitive protein (DELLA protein GAI), and plays a role in seed germination [72]. *Gh_A02G0111* is a homolog of *AT2G43410*, which encodes a flowering time control protein FPA in Arabidopsis [73]. *Gh_D03G1064* encodes a FRIGIDA-like protein that can pleiotropically increase lint yield; it is also significantly associated with SI [5]. The homologous gene of *Gh_D03G1064* in Arabidopsis is *FRI* (*AT4G00650*), which regulates flowering time in Arabidopsis [73–77].

GhFSN1 is a cotton NAC transcription factor that acts as a positive regulator to control secondary cell wall (SCW) formation in cotton fibers by activating downstream SCW-related genes, including *GhDUF231L1*, *GhKNL1*, *GhMYBL1*, *GhGUT1* and *GhIRX12* [66]. The candidate gene *Gh_A02G0101* also encodes a NAC protein (Table S7). The glucosyltransferases, Rab-like GTPase activators, and myotubularin (GRAM) domain gene *GhGRAM31* (*Ghir_D02G018120*) regulate fiber elongation. GhGRAM31 directly interacts with GhGRAM5 and GhGRAM35. GhGRAM5 also interacts with the transcription factor GhTTG1, and GhGRAM35 interacts with the transcription factors GhHOX1 and GhHD1 [67]. The candidate gene *Gh_A02G0094* also encodes the C2 and GRAM domain-containing protein At1g03370 (Table S7).

The above data demonstrate that most of the putative candidates of the six QTLs for LP and BW identified in this study were involved in regulating cotton fiber development. Most of the data obtained in this research are consistent with the findings of other studies, indicating that our results were reliable.

### Candidate gene expression profiles determine LP and BW

ZR014121 is an excellent high-yield but low-LP line. EZ60 is an early maturity line with high LP. The candidate gene expression profiles of the six QTLs for LP and BW in the two lines significantly differed (Fig. 6). Most candidate genes were highly expressed at the ovule developmental stage (0 DPA) in both ZR014121 and EZ60. Four key candidate genes were highly expressed at 5 DPA in ZR014121, including *Gh_A02G0095* (*BHLH113*, which might be involved in MYB-bHLH-WD40 complexes [33, 68]), *Gh_A02G0097* (*RGA3*), *Gh_A10G1158* (*CBDAS*), and *Gh_D03G1062* (*RBOHC*, which might be involved in ROS [70]). *Gh_A02G0114* (*ccdc94*) was significantly highly expressed at 15 DPA in EZ60. *Gh_A02G0101* (*NAC014*, which might be involved in SCW formation in cotton fibers [66]) was significantly highly expressed at 25 DPA in ZR014121.

Most genes were highly expressed at the ovule developmental stage, which demonstrates that these genes were highly active in this stage. The expression of four genes in ZR014121 after this stage was likely the main cause of high yield. These four genes, in addition to the other two highly expressed genes, *Gh_A02G0114* and *Gh_A02G0101*, were the key candidate genes of the six QTLs for LP and BW (Fig. 6). Although we were unable to determine whether the six genes represent the six QTLs, our findings indicate that they are the key genes regulating LP and BW and thus affecting cotton yield. These genes provide important genetic resources for studies of the lint regulation mechanism and improvements in cotton yield.

## Conclusions

Two RIL populations were constructed using the three excellent upland cotton lines ZR014121, CCRI60, and EZ60, which differ in fiber yield and quality traits. The RILs were genotyped by GBTS and phenotyped under four different environments; a GWAS was then conducted to identify useful yield-related QTLs. A total of 51 QTLs for LP and 49 QTLs for BW were identified, and these QTLs could explain 0.29–9.96% of the phenotypic variation in LP and 0.41–6.31% of the phenotypic variation in BW. There were six major and effective QTLs, three for LP and three for BW, and these could be used to breed cotton with high yield via molecular breeding approaches. A total of 108 putative candidate genes were identified in the six key QTLs, including genes that were positively related to the development of LP and BW, such as genes involved in gene transcription, protein synthesis, calcium signaling, phytohormone synthesis and signaling, and fiber synthesis-related polysaccharide metabolism. Seven of the candidate genes form a co-expression network. Six significantly highly expressed candidate genes after anthesis were important factors regulating cotton yield. These candidate genes will help clarify the molecular mechanisms underlying variation in LP and BW.

## Methods

### Plant materials and growth conditions

Three *G. hirsutum* lines ZR014121, CCRI60, and EZ60 were used as parents in this study, and they were bred at the Institute of Cotton Research, Chinese Academy of Agricultural Sciences. All of the three RIL lines we were authorized to use. EZ60 and ZR014121 were preserved in the National Germplasm Library (38 Huanghe Avenue, Anyang, Henan 455,000); their accession numbers were M116025 and ZM115357, respectively. CCRI60 is a variety. ZR014121 has high yield but low LP. EZ60 is an early maturity line with high LP. CCRI60 is an excellent cultivar with several desirable agronomic traits. Two RIL populations at the F_6:8_ generation in 2020 (at F_6:9_ in 2021), P-CCRI60 and P-EZ60 were constructed from crosses of ZR014121 × CCRI60 and ZR014121 × EZ60, respectively. P-CCRI60 consisted of 300 RILs, and P-EZ60 consisted of 200 RILs.

There were four factors in the field experiment: two years (2020 and 2021) and two locations (Anyang (36°05′N, 114°29′E), Henan Province, and Weixian (37°58′N, 115°16′E), Hebei Province, China(both of them are our experimental field)); these were each referred to as 20AY, 20WX, 21AY, and 21WX. To eliminate field effects, the experiment was conducted in a randomized incomplete block design with two replicates of each environmental factor. The parents and RILs were planted in rows with lengths of 3 m and widths of 0.8 m; the one control, CCRI60, had 20 rows. The lines were planted in April and sampled in September each year. Field management techniques followed those of regular breeding practices.

### Trait measurements

Two yield-related traits LP and BW were evaluated at each field location. The samples were prepared around September 20 each year. Thirty naturally opened bolls from the central part of plants (two bolls on each plant) of each line were randomly hand-harvested to calculate the BW (g) and gin the fiber. Fiber samples were separately weighed to calculate the LP (%). All statistical analyses, including correlations between traits, analysis of variance and significance analyses were conducted using IBM SPSS 22.0 software.

### GBTS

For genotyping, the young leaf tissues of the three parents ZR014121, CCRI60, and EZ60, and the RILs of the two populations, P-CCRI60 and P-EZ60, were sampled in July 2020. Genomic DNA was extracted from each sample using a modified cetyltrimethylammonium bromide method [78].

For GBTS, we used the Allegro Targeted Genotyping of NuGEN Technologies; the stable markers covering whole cotton genomes were selected from known markers obtained from the high-throughput sequencing results. To prevent the 3′-ends of the probes from overlapping with other known variable sites, the SNPs were tested in the parents and their F_1_ plants, and the polymorphic SNPs were used to design primers. DNA fragmentation, adapter ligation, target extension, and library amplification were performed following the instructions of various kits (NuGEN Technologies, San Carlos, California, USA). The libraries were tested using the most recently updated Illumina manufacturer’s instructions (Illumina, San Diego, CA, USA). Three replications of GBTS were performed on each sample.

After the SNP data were generated by BCFtools, the raw SNPs and Indels were screened using three parameters QUAL, RPB, and AC [(-e ‘%QUAL < 100); (RPB < 0.1, %QUAL < 100); (AC < 2, %QUAL < 100)’)]. The cover rate of each sequenced SNP was statistically analyzed using ‘samtools depth’. The SNPs with sequencing cover rates more than 10 times and without genotypes were considered to be genotypes consistent with those in the cotton reference genome; SNPs with sequencing cover rates less than one time and without genotypes were referred to as deletion genotypes. The two SNP quality control criteria were (1) call rate of a single locus and (2) call rate of an individual. The Perl soft program that we translated and edited was used to statistically analyze the quality control criteria. For the physical localizations of the SNP markers, the probe sequences of the SNPs were used to| perform local BLAST [79] queries against the *G. hirsutum* TM-1 reference genome [28, 52].

### GWAS

The high-quality SNPs determined from the whole study populations, P-CCRI60 and P-EZ60, were used to conduct a GWAS for LP and BW. Given the possibility of obtaining false-positive QTNs with low association frequencies, we selected QTNs with LOD > 3 as stable QTNs in subsequent analyses. The software 3VmrMLM version 1.0 [80] was used to perform GWAS with the following settings: method = ‘Multi_env’; fileKin = NULL; filePS = NULL; PopStrType = ‘Q’; fileCov = NULL; SearchRadius = 20; svpal = 0.01; DrawPlot = TRUE; Plotformat = ‘pdf’; and Chr_name_com = NULL. We obtained significant and suggested main-effect QTNs, significant, as well as suggested QEIs. The significant QTNs were selected by Bonferroni correction, and the critical *P*-value was 0.05/m, where m is the number of tests or markers, and suggested QTNs were identified as those with LOD ≥ 3.0. Significant QEIs were selected by Bonferroni correction; the critical *P*-value was 0.05/m, where m is the number of tests or markers, and suggested QEIs were identified as those with LOD ≥ 3.0 using default parameters [80].

### Prediction and identification of candidate genes

We defined the flanking 200-Kb regions of the QTNs as the same QTL and merged the overlapping QTLs to confirm the number of QTLs [81]. Potential candidate genes were confirmed based on gene annotations in the *G. hirsutum* TM-1 genome [28, 52]. All the candidate genes were subjected to Gene Ontology [82] enrichment analysis and Kyoto Encyclopedia of Genes and Genomes [83–85] analysis. The interaction network of candidate genes was inferred by constructing a PPI network using the STRING database [52]. The network analysis was conducted using Cytoscape 3.7.2.

### RNA sequencing and gene expression profiles of the QTL candidates

The ovules/fibers of EZ60 and ZR014121 were sampled at 0, 5, 10, 15, 20, and 25 days post-anthesis (DPA). The total RNAs were extracted using the mirVana™ miRNA Isolation Kit (Ambion) according to the manufacturer’s instructions. Three biological replicates were performed for each sample. The Illumina PE libraries were sequenced on the HiSeqTM2500 (Illumina) platform. Raw reads were filtered using Trimmomatic-0.39 [86], and the clean reads were mapped to the reference genome [87] using STAR-2.7.9a [88]; the abundances of transcripts were quantified using RSEM-1.2.26 [89]. Differentially expressed genes (DEGs) were identified using DESeq2-1.30.1 according to the following criteria: padj < 0.05 and log_2_^(FoldChange)^ > 1 DESeq2-1.30.1 [90]. Hierarchical cluster analysis of DEGs was conducted to measure expression levels. The expression profiles of every candidate gene were used to preliminarily identify LP-related and BW-related genes.

## Supplementary Information

**Additional file 1:Table S1.** The result of GBTS

**Additional file 2:Table S2.** The results of sample genotyping

**Additional file 3:Table S3.** The result of 3VmrMLM: QEI

**Additional file 4:Table S4.** The identified QTLs

**Additional file 5:Table S5.** The identified QTLs overlapped with the reported QTLs

**Additional file 6:Table S6.** The identified new QTLs

**Additional file 7:Table S7.** All candidate genes of the 6 key QTLs

**Additional file 8:Table S8.** Annotations of the candidate genes of the six QTLs for BW and LP

**Additional file 9:Table S9.** KEGG annotations of the candidate genes of the QTLs for LP

**Additional file 10:Table S10.** KEGG annotations of the candidate genes of the QTLs for BW

**Additional file 11:Table S11.** The expression levels of the candidate genes 

## Data Availability

The datasets generated and/or analyzed during the current study are available in the NCBI repository, **[** https://www.ncbi.nlm.nih.gov/bioproject/906276**]** [Accession number: PRJNA906276].
